# Research on Molecular Structure and Electronic Properties of Ln^3+^ (Ce^3+^, Tb^3+^, Pr^3+^)/Li^+^ and Eu^2+^ Co-Doped Sr_2_Si_5_N_8_ via DFT Calculation

**DOI:** 10.3390/molecules26071849

**Published:** 2021-03-25

**Authors:** Ziqian Yin, Meijuan Li, Jianwen Zhang, Qiang Shen

**Affiliations:** 1 State Key Laboratory of Advanced Technology for Materials Synthesis and Processing, Wuhan University of Technology, Wuhan 430070, China; yinziqian1996@163.com (Z.Y.); elliott@whut.edu.cn (J.Z.); 2 School of Chemistry, Chemical Engineering and Life Sciences, Wuhan University of Technology, Wuhan 430070, China

**Keywords:** first principles, density functional theory calculations, electronic structure, photoluminescence material, Sr_2_Si_5_N_8_:Eu^2+^

## Abstract

We use density functional theory (DFT) to study the molecular structure and electronic band structure of Sr_2_Si_5_N_8_:Eu^2+^ doped with trivalent lanthanides (Ln^3+^ = Ce^3+^, Tb^3+^, Pr^3+^). Li^+^ was used as a charge compensator for the charge imbalance caused by the partial replacement of Sr^2+^ by Ln^3+^. The doping of Ln lanthanide atom causes the structure of Sr_2_Si_5_N_8_ lattice to shrink due to the smaller atomic radius of Ln^3+^ and Li^+^ compared to Sr^2+^. The doped structure’s formation energy indicates that the formation energy of Li^+^, which is used to compensate for the charge imbalance, is the lowest when the Sr2 site is doped. Thus, a suitable Li^+^ doping site for double-doped lanthanide ions can be provided. In Sr_2_Si_5_N_8_:Eu^2+^, the doped Ce^3+^ can occupy partly the site of Sr_1_^2+^ ([SrN_8_]), while Eu^2+^ accounts for Sr_1_^2+^ and Sr_2_^2+^ ([SrN_10_]). When the Pr^3+^ ion is selected as the dopant in Sr_2_Si_5_N_8_:Eu^2+^, Pr^3+^ and Eu^2+^ would replace Sr_2_^2+^ simultaneously. In this theoretical model, the replacement of Sr^2+^ by Tb^3+^ cannot exist reasonably. For the electronic structure, the energy level of Sr_2_Si_5_N_8_:Eu^2+^/Li^+^ doped with Ce^3+^ and Pr^3+^ appears at the bottom of the conduction band or in the forbidden band, which reduces the energy bandgap of Sr_2_Si_5_N_8_. We use DFT+U to adjust the lanthanide ion 4f energy level. The adjusted 4f-CBM of Ce_Sr1_Li_Sr1_-Sr_2_Si_5_N_8_ is from 2.42 to 2.85 eV. The energy range of 4f-CBM in Pr_Sr1_Li_Sr1_-Sr_2_Si_5_N_8_ is 2.75–2.99 eV and its peak is 2.90 eV; the addition of Ce^3+^ in Eu_Sr1_Ce_Sr1_Li_Sr1_ made the 4f energy level of Eu^2+^ blue shift. The addition of Pr^3+^ in Eu_Sr2_Pr_Sr2_Li_Sr1_ makes part of the Eu^2+^ 4f energy level blue shift. Eu^2+^ 4f energy level in Eu_Sr2_Ce_Sr1_Li_Sr1_ is not in the forbidden band, so Eu^2+^ is not used as the emission center.

## 1. Introduction

Red fluorescent materials are essential parts of improving the color rendering index in phosphor-converted WLEDs (pc-WLEDs) and have high application value. With rare-earth ions as activating ions, as a representative of the matrix of red phosphors, Sr_2_Si_5_N_8_ alkaline earth metal silicon nitride has been extensively studied in recent years [1,2,3]. When Eu^2+^ is used as the activating ion, the luminous intensity is the highest. Simultaneously, Sr_2_Si_5_N_8_:Eu^2+^ has become the representative of commercial red fluorescent materials because of its outstanding fluorescence performance in all aspects [4]. However, the main problem is that Sr_2_Si_5_N_8_:Eu^2+^ is sensitive to temperature, and Sr_2_Si_5_N_8_:Eu^2+^ luminous intensity is significantly reduced when the temperature is higher. Considering that M_2_Si_5_N_8_ is a layered or similar layered structure, its openness is relatively high so that the above problems can be solved through component engineering [5].

The coordination environment, electronic structure, and morphological characteristics of the Eu^2+^ ion are several vital factors that affect phosphors’ luminescence performance. They determine the luminescence characteristics by indirectly changing the degree of crystal field splitting (CFS) [6], nephelauxetic effect (NE), the highest and lowest 5d energy level splitting [7,8]. For example, from both experiments and calculations Li [9] and Bulloni [10] proved that Ca^2+^ partially replaced Eu^2+^ in Sr_2_Si_5_N_8_ matrix’s emission peaks, which tended to appear red-shifted in Eu^2+^ occupied eight coordination sites, though its stability was reduced. Liu used Ba^2+^ to replace partial Sr^2+^ in Sr_2_Si_5_N_8_, after the substitution, the emission peak was blue-shifted. As Eu^2+^ in the ten-coordinate structure is more stable than the eight-coordinate structure, its thermal stability is improved. Chen [11] performed a doping modification based on Sr_2_Si_5_N_8_:Eu^2+^. In Sr_2_Si_5_N_8_:Eu^2+^, part of Al^3+^ is used to replace Si^4+^. As the Al-N bond length is longer than that of Si-N, the bond length between Eu^2+^ and surrounding N^3−^ is shorter, the crystal field intensity increases, and the emission peak position is red-shifted. Wang [12] used partial AlO^+^ instead of SiN^+^, and the effects of the increase in the crystal field and the increase in electronegativity cancelled each other out, rendering the peak position unchanged, but the thermal stability and strength increased. In Rb_3_Ysi_2_O_7_:Eu^2+^ system, the weak covalent interaction of Eu^2+^ and O^2−^ prevented Eu^2+^ from showing red emission [13]. The above-mentioned previous studies had found that different activating ions and ligand sites affected the energy level distribution of the activated ions and f orbitals, thereby affecting the luminescence performance.

Doping with more than one lanthanide ion can make up for the deficiency of one lanthanide ion doping. For example, Li [14] successfully introduced Gd^3+^/Er^3+^/Lu^3+^ into Bi_2_Mo_6_ to enhance its photocatalytic performance. Tang [15] introduced Ce^3+^ and Tb^3+^ into Na_3_SrMg_11_(PO_4_)_9_. There are relatively few reports on the lanthanide Eu^2+^ doped with M_2_Si_5_N_8_ as the base material and further doped with another lanthanide. The study found that Tb^3+^ and Eu^2+^ co-doped Sr_2_Si_5_N_8_ has a 20% increase in emission intensity [16]. Therefore, we want to systematically study the changes in the molecular structure and luminescence properties of Eu^2+^ and other Ln^3+^ co-doped systems. Among many lanthanides, the excitation spectrum of Pr^3+^4f-5d is relatively simple [8]. In the [Xe] (near nuclear pseudopotential electron) 4f_1_5d_1_ configuration during the excitation, Pr^3+^ has only one 4f energy level, which can occupy two different electrons. Tb’s advantage is that in 4f_7_5d, the 4f_7_[^8^S_7/2_] energy level is relatively stable, and the next higher 4f_7_[^6^P_J_]5d_1_ energy level is about 3.5–4.0 eV higher. Therefore, it can be observed that 4f_8_–4f_7_(^8^S_7/2_)5di turns into an isolated state. Ce^3+^ is widely used as an activating ion in various fluorescent systems: Lu_3_Al_5_O_12_:Ce^3+^ [17], LaSi_6_N_11_:Ce^3+^ [18], Tb_3_Al_5_O_12_:Ce^3+^ [19], In summary, so we prefer to use any one of Ce^3+^, Pr^3+^, Tb^3+^ and Eu^2+^ doping for the Sr_2_Si_5_N_8_ matrix to explore the changes in molecular structure and properties.

Li [20] used Ce^3+^ and Li^+^ co-doping to replace two Sr^2+^, among which Li^+^ was used as a charge compensator for the charge imbalance caused by the partial replacement of Sr^2+^ by Ln^3+^. Li^+^ was widely used in phosphor doping such as CaLiAl_3_N_4_ [21], Sr_4_LiAl_11_N_14_ [22], Li_2_Ca_2_Mg_2_Si_2_N_6_ [23].

To realize the fundamental principal research on the luminescence characteristics, Fang [24] used the first principles to calculate the molecular structure and energy band structure of M_2_Si_5_N_8_ (M = Ca, Sr). Shen [25] studied the band structure of Sr_2_Si_5_N_8_:Eu^2+^ through first-principles calculations and combined experiments to reveal the mechanism of luminescence. Density functional theory (DFT) based on first-principles ideas has been successfully applied to the study of microscopic particle systems. In this paper, combined with previous studies, first-principles calculations are used to study the model of Ce^3+^, Pr^3+^, Tb^3+^, respectively, with Li^+^ co-doped Sr_2_Si_5_N_8_ matrix system and Ce^3+^/Pr^3+^/Tb^3+^, respectively, with Eu^2+^, Li^+^ three types of ions co-doped Sr_2_Si_5_N_8_ matrix. The optimized structural parameters of the co-doping model for different ion species and sites are presented. We calculate the energy band and density of states of varying doping systems to analyze the electronic structure.

## 2. Results and Discussion

### 2.1. Structures Distortion of Doped Models

In Table 1, a (Å), b (Å), c (Å) are the three sets of edge lengths of the unit cell. α, β, γ**/**(°) are, respectively, the angle between b and c; a and c; a and b. Polyhedral volume (Å^3^) is the coordination polyhedron volume of Ln and N. Distortion index (Å) is the distance a ligand moves after the d/f orbital energy level splits and stabilizes, which reflects distortion effect. Distortion effect: For transition element/rare earth element ions with a high coordination number (>6), high-spin d/f orbitals and low-spin d/f orbitals are unstable in regular polyhedrons, which will cause these d/f orbits to undergo further splitting in energy, in order to stabilize the ion, causing the coordination relationship to deviate from the symmetry of the regular polyhedron. Effective coordination number means that due to the regular coordination polyhedron’s structural distortion, the bond length between the ligand and the central atom changes, resulting in non-integer coordination.

The structure of Tb_Sr2_Li_Sr2_-Sr_2_Si_5_N_8_ cannot converge after optimization. This model is unlikely to exist in the actual doping process, so subsequent calculations are not considered. The ionic radius of Sr^2+^ (1.18 Å), Eu^2+^ (1.17 Å), Ce^3+^ (1.02 Å), Pr^3+^ (0.99 Å), Tb^3+^ (0.92 Å), Li^+^ (0.68 Å) [26] (regardless of the coordination number) decreases from left to right; Eu^2+^, Ce^3+^/Li^+^, Pr^3+^/Li^+^ replace Sr^2+^ in turn. Due to the cationic ligand’s volume coarctation, the lattice constant and unit cell volume will be slightly smaller. The volume of the doped system is smaller than that of undoped lanthanide ions. Sr_2_Si_5_N_8_ volume, unit cell volume and doped ion radius are positively correlated. The average bond length between the lanthanide ion and N becomes shorter, making the bond between the lanthanide ion and the surrounding N stronger. The structure is more compact, the crystal field strength increases, and there will be a redshift tendency. Comparing the system in which the same lanthanide ion replaces eight-coordinate Sr^2+^ and ten-coordinate Sr^2+^, we find that the distortion degree of eight-coordinate Sr^2+^ is greater than that of ten-coordinate Sr^2+^. The formation of an eight-coordinate structure will produce a stronger electron cloud. The nephelauxetic effect (NE) produces a centroid shift, which has a synergistic effect with the above redshift. However, for trivalent lanthanide ions doped with the same coordination number, the different Li^+^ sites have almost no effect on the structure, which is only used to balance the charge.

**Figure 1:**
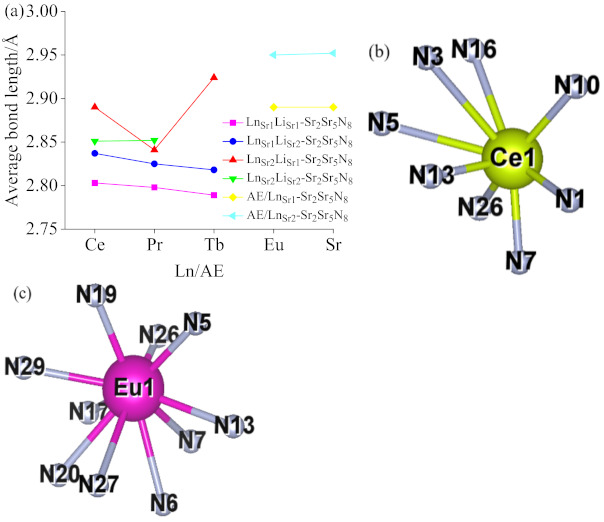
(**a**) Average bond length of [LnN_8_] and [LnN_10_]. (**b**) Structure diagram of [CeN_8_]. (**c**) Structure diagram of [EuN_10_].

Figure 1a shows the average bond length of [LnN_8_] and [LnN_10_], where AE represents the average bond length of [SrN_8_] and [SrN_10_] ligands in Sr_2_Si_5_N_8_ primitive unit cells. It can be seen from the figure that the average bond length decreases with the decrease in the ion radius. Ln_Sr2_Li_Sr1_-Sr_2_Si_5_N_8_ has the most extended bond length on average, and Ln_Sr1_Li_Sr1_-Sr_2_Si_5_N_8_ is the shortest. Figure 1b is a schematic diagram of Ce_Sr1_Li_Sr1_-Sr_2_Si_5_N_8_ [CeN_10_] and Eu_Sr1_-Sr_2_Si_5_N_8_ [EuN_10_]. Atoms (10 N) are selected from the 32 N atoms to form a ten-coordinate polyhedron in the figure.

#### 2.1.1. Ln-Li Distance and [SrN] Coordination Polyhedron Parameters of Sr_2_Si_5_N_8_:Ln^3+^/Li^+^

**Figure 2:**
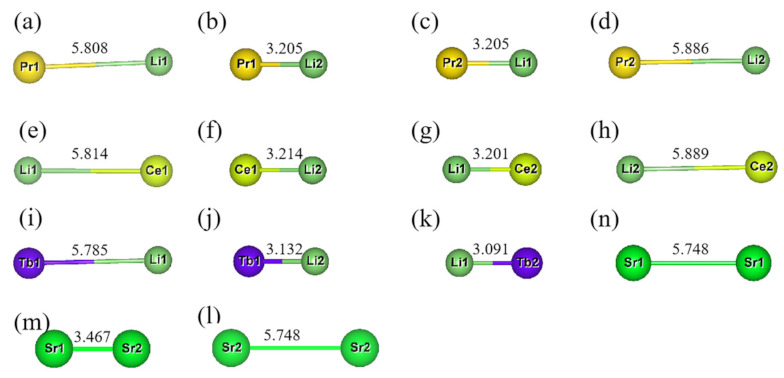
The distance change after Ln^3+^ and Li^+^ replace Sr_1_ and Sr_2_ in doped Sr_2_Si_5_N_8_ (**a**–**k**) Pr_Sr1_Li_Sr1_-Sr_2_Si_5_N_8_, Pr_Sr1_Li_Sr2_-Sr_2_Si_5_N_8_, Pr_Sr2_Li_Sr1_-Sr_2_Si_5_N_8_, Pr_Sr2_Li_Sr2_-Sr_2_Si_5_N_8_, Ce_Sr1_Li_Sr1_-Sr_2_Si_5_N_8_, Ce_Sr1_Li_Sr2_-Sr_2_Si_5_N_8_, Ce_Sr2_Li_Sr1_-Sr_2_Si_5_N_8_, Ce_Sr2_Li_Sr2_-Sr_2_Si_5_N_8_, Tb_Sr1_Li_Sr1_-Sr_2_Si_5_N_8_, Tb_Sr1_Li_Sr2_-Sr_2_Si_5_N_8_, Tb_Sr2_Li_Sr1_-Sr_2_Si_5_N_8_; (**n**–**l**) is the Sr-Sr distance before doping.

**Figure 3:**
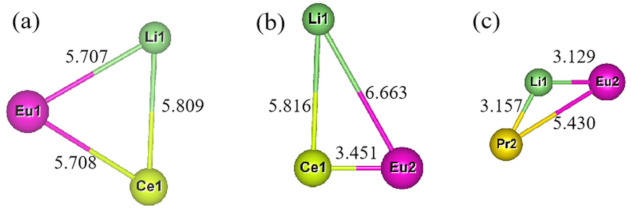
(**a**–**c**): Ln-Li, Eu-Li, Eu-Ln’s distance diagram after Eu_Sr1_Ce_Sr1_Li_Sr1_-Sr_2_Si_5_N_8_, Eu_Sr2_Ce_Sr1_Li_Sr1_-Sr_2_Si_5_N_8_, Eu_Sr2_Pr_Sr2_Li_Sr1_-Sr_2_Si_5_N_8_ doped with Sr_2_Si_5_N_8_ matrix.

Figure 2 and Figure 3 describes the distance between Ln^3+^ and Li^+^. In the Sr_2_Si_5_N_8_ matrix, the selected doping (Sr) site is the same distance as Sr_1_-Sr_1_ and Sr_2_-Sr_2_, before being replaced by Ln^3+^/Li^+^, which has a distance of 5.748 Å; the distance is 3.467 Å between Sr_1_-Sr_2_. When Ln^3+^ and Li^+^ are doped to replace Sr_1_, in the order of Pr^3+^, Ce^3+^, Tb^3+^, the Ln-Li distances increased by 0.06 Å, 0.066 Å, 0.037 Å, respectively, and the degree of distortion was 1.05%, 1.14% and 0.64%. The overall deviation is not significant. When Ln^3+^/Li replaces different sites, the degree of distortion is always above 7.56%. From the perspective of the degree of lattice distortion, it is unlikely to occur in actual situations. When Ln^3+^/Li^+^ is doped to replace Sr_2_, the distortion degree of Pr_Sr2_Li_Sr2_-Sr_2_Si_5_N_8_ and Ce_Sr2_Li_Sr2_-Sr_2_Si_5_N_8_ is about 2.40%. After Ln_Sr1_Li_Sr_-Sr_2_Si_5_N_81_ and Ln_Sr2_Li_Sr2_-Sr_2_Si_5_N_8_ are doped to replace the Sr site, the distance between Ln-Li becomes longer, while Ln_Sr1_Li_Sr2_ and Ln_Sr2_Li_Sr1_ have shorter distances than that before doping.

**Figure 4:**
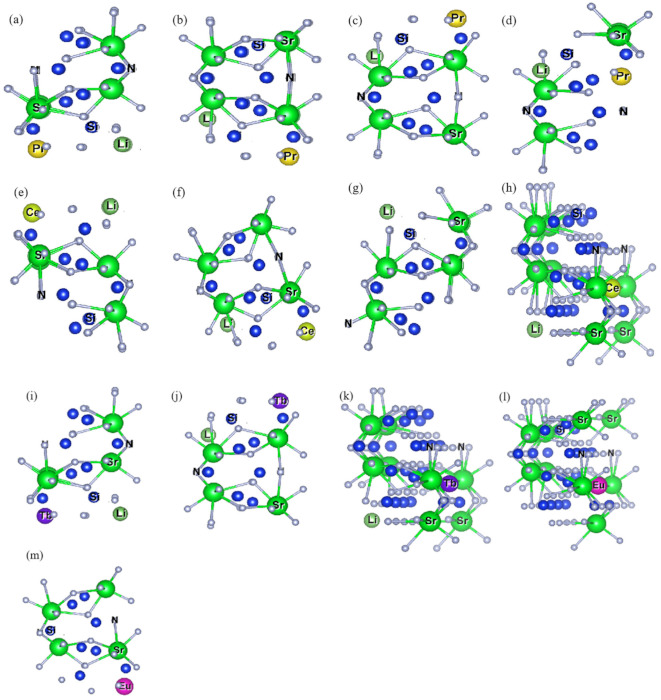
[SrN] coordination polyhedron diagram after substitution of Ln^3+^/Li^+^ for Sr_1_ and Sr_2_ sites in Sr_2_Si_5_N_8_ (**a**–**m**) Pr _Sr1_Li_Sr1_-Sr_2_Si_5_N_8_, Pr_Sr1_Li_Sr2_-Sr_2_Si_5_N_8_, Pr_Sr2_Li_Sr1_-Sr_2_Si_5_N_8_, Pr_Sr2_Li_Sr2_-Sr_2_Si_5_N_8_, Ce_Sr1_Li_Sr1_-Sr_2_Si_5_N_8_, Ce_Sr1_Li_Sr2_-Sr_2_Si_5_N_8_, Ce_Sr2_Li_Sr1_-Sr_2_Si_5_N_8_, Ce_Sr2_Li_Sr2_, Tb_Sr1_Li_Sr1_-Sr_2_Si_5_N_8_, Tb_Sr1_Li_Sr2_-Sr_2_Si_5_N_8_, Tb_Sr2_Li_Sr1_-Sr_2_Si_5_N_8_, Eu_Sr1_-Sr_2_Si_5_N_8_, Eu_Sr2_-Sr_2_Si_5_N_8_.

Table 2 is the detailed description of Figure 4. In Table 2, we can see that when Ln doping replaces the site, [SrN_10_] tends to appear with two coordination types, one has an effective coordination number greater than 6.707 Å, and the other is less than 6.707 Å (6.707 Å is undoped [SrN_10_] effective coordination number). The effective coordination number of [SrN_10_] increases, while the effective coordination number of [SrN_8_] decreases. When Ln^3+^ doping replaces the Sr_2_ site, it is similar to Ln^3+^ substitution doping of the Sr_1_ site. [SrN_8_] tends to have two effective coordination numbers: the effective coordination number of [SrN_8_] increased, and the effective coordination number of [SrN_10_] decreased. The above phenomenon showed that after the structure optimization of Ln doping to replace Sr sites, the effective coordination number of [SrN] in other Sr sites is reduced with the same coordination number as doping to replace Sr is reduced. Combining with three factors: [LnN] coordination polyhedron structure, Ln-Li distance and [SrN] coordination polyhedron structure, the Ln_Sr1_Li_Sr1_-Sr_2_Si_5_N_8_ doped-model can exist reasonably.

#### 2.1.2. Lattice Constant and [LnN] Ligand Parameters of Sr_2_Si_5_N_8_:Ln^3+^/Li^+^/Eu^2+^

In Table 3, Ln^3+^/Eu^2+^ is the coordination polyhedron information of the ion and N. Except for the six ligand structures with three double lanthanide ions substituted for the Sr site, the other structures are not below the theoretical values. Only the following three doping models are Eu^2+^ and Ce^3+^ to replace Sr_1_, Eu^2+^ to replace Sr_2_, and Ce^3+^ to replace Sr_1_, Eu^2+^ replaces Sr_2_, Pr^3+^ replaces Sr_2_ in line with the actual structure. Neither Tb^3+^ nor Eu^2+^ co-doped systems are desirable. The unit cell volume and ligand structure of the co-doped system did not change significantly from the single-doped system.

#### 2.1.3. Ln-Li-Eu distance and [SrN] Coordination Polyhedron Parameters of Sr_2_Si_5_N_8_:Ln^3+^/Li^+^/Eu^2+^

**Figure 5:**
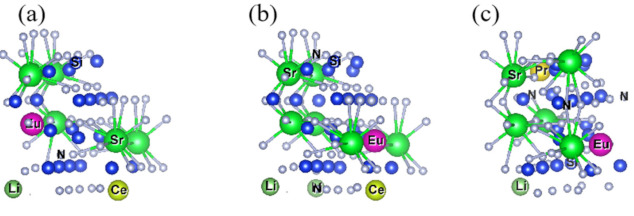
(**a**–**c**): [SrN] Coordination Polyhedron Diagram after Eu_Sr1_Ce_Sr1_Li_Sr1_-Sr_2_Si_5_N_8_, Eu_Sr2_Ce_Sr1_Li_Sr1_-Sr_2_Si_5_N_8_, Eu_Sr2_Pr_Sr2_Li_Sr1_-Sr_2_Si_5_N_8_ doped with Sr_2_Si_5_N_8_ matrix.

After screening more than 30 models, a total of three models may exist stably after structure optimization. According to the order of Figure 4a–c, the distances before optimization of Ln-Li, Ln-Eu and Eu-Li are 5.748 Å, 5.748 Å, 5.748 Å; 5.748 Å, 3.467 Å, 6.713 Å; 3.467 Å, 5.748 Å, 3.467 Å. Among them, the distance between (a) and (b) does not change obviously before and after convergence, which is less than 0.1% compared with the original Sr-Sr distance. In summary, Combining Figure 5 and Table 4, we can draw the following conclusions: Eu_Sr1_Ce_Sr1_Li_Sr1_ and Eu_Sr2_Ce_Sr1_Li_Sr1_ can exist stably after Eu^2+^/Ce^3+^/Li^+^ co-doped with Sr_2_Si_5_N_8_, without considering the formation energy conditions. In the Eu_Sr2_Pr_Sr2_Li_Sr1_-model, there are three distance types: Ln-Eu, Ln-Li, and Eu-Li. Compared with the original Sr sites, the distance changes are 5.6%, 8.9%, and 9.7%, respectively. In the Eu^2+^/Tb^3+^/Li^+^-Sr_2_Si_5_N_8_ model, the ionic radius of Tb^3+^ is too small, resulting in excessive structural distortion and difficulty in optimization convergence, so its structure cannot exist stably.

### 2.2. Formation Energy of Doped Models

**Figure 6:**
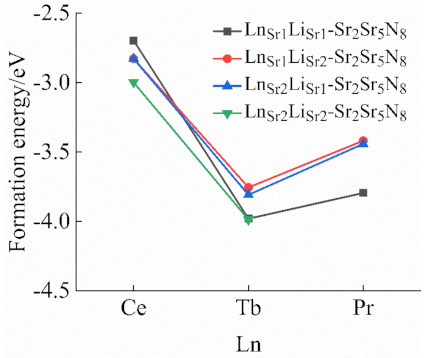
Formation energies of Ln^3+^/Li^+^ elements co-doped Sr_2_Si_5_N_8_.

Figure 6 is the formation energy diagram of Ce^3+^ (Tb^3+^, Pr^3+^)/Li^+^ co-doped Sr_2_Si_5_N_8_ with eight-coordinate (Sr_1_) and ten-coordinate (Sr_2_). From the definition of formation energy, the lower formation energy value means the target product is easier to form. Among all the values, Ce^3+^ and Li^+^’s formation energy co-doped in eight-coordinate and ten-coordinate systems, respectively, is the lowest. Pr^3+^ and Li^+^ co-doped together to replace eight-coordinate Sr has the highest formation energy. For Ce^3+^ and Tb^3+^, Ce^3+^ (Tb^3+^) and Li^+^ are, respectively, doped at the same Sr site to form lower energy. Pr and Li co-doped to replace ten-coordinate Sr^2+^ has the lower formation energy, and for co-doped to replace eight-coordinate Sr^2+^, the formation energy is the highest. Three kinds of lanthanide ions doping to replace Sr_2_ are easier to generate in theory. The formation energy of Li^+^ is lower when it is at the Sr_2_ site, so the fixed Li^+^ doping replaces Sr_2_^2+^. Ce^3+^ (Tb^3+^, Pr^3+^)/Li^+^/Eu^2+^ co-doped Sr_1_ and Sr_2_ of Sr_2_Si_5_N_8_, in which Li^+^ is fixedly doped instead of Sr_1_^2+^. Eu_Sr1_Ce_Sr1_Li_Sr1_-Sr_2_Si_5_N_8_, Eu_Sr2_Ce_Sr1_Li_Sr1_-Sr_2_Si_5_N_8_ and Eu_Sr2_Pr_Sr2_Li_Sr1_-Sr_2_Si_5_N_8′_s formation energy levels are −1.2 eV, −5.2 eV, −5.18 eV. Compared with Eu_Sr2_Pr_Sr2_Li_1_-Sr_2_Si_5_N_8_, Sr_2_Si_5_N_8_:Pr^3+^/Li^+^ has a lower formation energy at around −2.8 eV. The formation energy of Sr_2_Si_5_N_8_:Ce^3+^/Li^+^/Eu^2+^ is 1.2 eV lower than Sr_2_Si_5_N_8_:Ce^3+^/Li^+^, so Eu_Sr1_Ce_Sr1_Li_Sr1_-Sr_2_Si_5_N_8_, Eu_Sr2_Ce_Sr1_Li-Sr_2_Si_5_N_8_ could exist in theory.

### 2.3. Band Structures and Density of States

**Figure 7:**
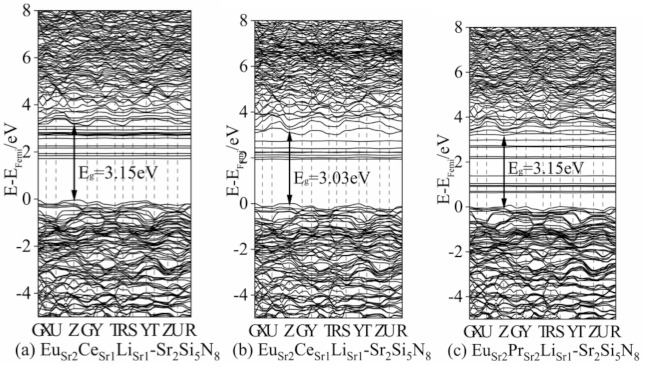
Band structures of doped structures: (**a**) Eu_Sr1_Ce_Sr1_Li_Sr1_-Sr_2_Si_5_N_8_; (**b**) Eu_Sr2_Ce_Sr1_Li_Sr1_-Sr_2_Si_5_N_8_; (**c**) Pr_Sr2_Eu_Sr2_Li_Sr1_-Sr_2_Si_5_N_8_.

We first calculated the ground state energy band and state density of Eu^2+^ single-doped Sr_2_Si_5_N_8_ and Tb^3+^ (Ce^3+^, Pr^3+^)/Li^+^ co-doped Sr_2_Si_5_N_8_ systems, as shown in Figure 7, for our subsequent calculations of Eu^2+^/Ce^3+^ (Tb^3+^, Pr^3+^)/Li^+^ ion co-doping, which provides a basis for comparison. The calculated bandgap of Sr_2_Si_5_N_8_:Eu_2_^2+^ is 3.21 eV, which is slightly smaller than the experimental bandgap because the approximate processing of the DFT exchange-correlation term causes the bandgap to become narrower [27]. In the (a–l) ground-state band structure diagram, their direct bandgaps are Ce_Sr1_Li_Sr1_-Sr_2_Si_5_N_8_: 3.03 eV, Ce_Sr1_Li_Sr2_-Sr_2_Si_5_N_8_: 3.15 eV, Ce_Sr2_Li_Sr1_-Sr_2_Si_5_N_8_:Ce_Sr2_Li_Sr1_-Sr_2_Si_5_N_8_:Sr_2_Si_5_N_8_: 3.09 eV, Pr_Sr1_Li_Sr1_-Sr_2_Si_5_N_8_: 2.97 eV, Pr_Sr1_Li_Sr2_-Sr_2_Si_5_N_8_: 3.27 eV, Pr_Sr2_Li_Sr1_-Sr_2_Si_5_N_8_: 3.27 eV, Pr_Sr2_Li_Sr2_-Sr_2_Si_5_N_8_: 3.30 eV, Tb_Sr1_Li_Sr1_-Sr_2_Si_5_N_8_: 3.30 eV, Tb_Sr1_Li_Sr2_-Sr_2_Si_5_N_8_: 3.27 eV, Tb_Sr1_Li_Sr2_-Sr_2_Si_5_N_8_: 3.24 eV. The high symmetry point G (reciprocal space), has the lowest energy at conduction band minimum (CBM), while some high symmetry points (Z) have the lowest CBM, and the highest valence band maximum (VBM). The lowest point of energy is between Z and G (Figure S1, Supporting Information). Due to the large energy gap between VBM and CBM (0.3 eV), the transition from VBM to CBM is an indirect bandgap transition. The direct bandgaps of (a–k) are 0.51 eV, 0.78 eV, 0.87 eV, 0.57 eV; 0.39 eV, 0.18 eV, 0.18 eV, 0.27 eV, 1.98 eV, 2.07 eV, 2.01 eV (Figure S1, Supporting Information); (a–k)’s indirect bandgaps are 3.03 eV, 3.15 eV, 3.24 eV, 3.09 eV, 2.97 eV, 3.27 eV, 3.27 eV, 3.30 eV, 3.30 eV, 3.27 eV, 3.24 eV. We compare four data from each of the same lanthanide elements. Excluding Pr_Sr2_Li_Sr2_-Sr_2_Si_5_N_8_, the bandgap is 3.30 eV, and the other five Ln_n_Li_n_-Sr_2_Si_5_N_8_ are all about 3 eV. Except for Pr_Sr2_Li_Sr2_-Sr_2_Si_5_N_8_, (f–h)’s 4f is closed to the CBM, the 4f energy levels of the ground-state lanthanide ions are distributed near the Fermi level (set VBM to 0), which is the premise of the lanthanide ion itself as the luminescence center. (a) Ce_Sr1_Li_Sr1_-Sr_2_Si_5_N_8_ and (e) Pr_Sr1_Li_Sr1_-Sr_2_Si_5_N_8_ have the smallest band gaps in their respective doping models, indicating that these two doping models have a high peak in the excited state and better luminous performance.

The states diagram’s density shows that the main components of VBM are 2p of N, 3s, 3p of Si, and CBM is mainly composed of 4f energy level of La, 5d, 5s orbitals of Sr and 3p, 3s of Si. Eu 6s, Eu 5p, Ce 6s, Ce 5p, Pr 6s, Pr 5p, Tb 6s and Tb 5p are minor in their contributions. The (Partial Density of State, PDOS) of Ce, Sr, N, Si in Ce_Sr1_Li_Sr1_-Sr_2_Si_5_N_8_ are the same as the four PDOS in Sikander Azam’s calculation about Sr_2_Si_5_N_8_:Ce^3+^ [28]. Figure 7a has strong peaks at 2.04 eV and 2.46 eV, Figure S1b has approximately the same values at 2.07 eV, 2.10 eV, 2.25 eV, and 2.34 eV, while Figure S1c has a higher peak at 2.31 eV. Figure S1d has peaks of similar intensity at 2.25 eV, 2.43 eV, and 2.49 eV. The 4f energy level of Figure S1f–h is between 2.28 and 2.52 eV, and the 4f peak value of Pr^3+^ in Figure S1e is very high. If Pr^3+^ is the luminous center, the luminous intensity is much higher than Figure S1f–h. The 4f of Tb^3+^ in Figure S1j has a higher peak intensity at 1.17 eV, which has a good potential for activating ions.

Figure 7 shows the energy bands and state density of the three kinds of triple-doped ions systems, Eu_Sr1_Ce_Sr1_Li_Sr2_-Sr_2_Si_5_N_8_, Eu_Sr2_Ce_Sr1_Li_Sr2_-Sr_2_Si_5_N_8_, Pr_Sr2_Eu_Sr2_Li_Sr2_-Sr_2_Si_5_N_8_. In Eu_Sr1_Ce_Sr1_Li_Sr2_-Sr_2_Si_5_N_8_, Eu^2+^ is the main component in the forbidden band. Ce^3+^ is close to the bottom of the conduction band. Eu_Sr2_Ce_Sr1_Li_Sr2_-Sr_2_Si_5_N_8_ is in the conduction band and has low intensity. Therefore, among the lanthanide ions of this system, only Ce^3+^ is the luminescence center. For Pr_Sr2_Eu_Sr2_Li_Sr2_-Sr_2_Si_5_N_8_, the band distribution is relatively dense. The 5d electrons in the excited state may produce multi-level transitions [29].

#### Determination of DFT^+^U Parameters of Each System

In Table 5, as the value of U_eff_ increases from 0 to 8 eV, when Eu^2+^ is equal to 6 eV, the 4f electron orbital of Eu^2+^ appears at the top of the valence band. When U_eff_ = 8 eV, the filled state 4f orbital has wholly entered the valence band, and the energy level is about −1 eV (set the top of the valence band as the Fermi level, that is, E_f_ = 0 eV). When U_eff_ = 4 eV, Sr_2_Si_5_N_8_:Eu_Sr1_^2+^ and Sr_2_Si_5_N_8_:Eu_Sr2_^2+^’s 4f–CBM energy difference is 2.22 eV and 2.23 eV, respectively, according to the energy wavelength conversion formula:

(1) $$E=\frac{hkC}{\lambda }$$

In the above formula, *E* (energy)—eV, *k* (Planck’s constant) = 6.63 × 10^−34^ J·s, *k* = 1.6 × 10^−19^ J/eV, *C* (speed of light) = 3 × 10^17^ nm/s, *λ* (wavelength)—nm. The parameters can be obtained in the following formula:

(2) $$\lambda =\frac{1240}{E}$$

**Figure 8:**
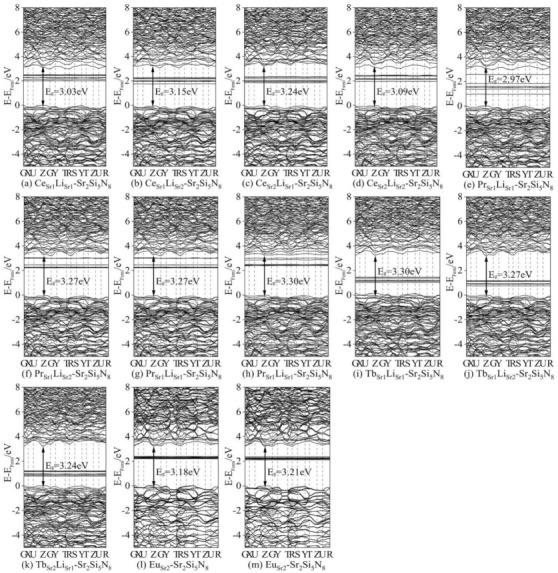
Band structures of doped structures: (**a**) Ce_Sr1_Li_Sr1_-Sr_2_Si_5_N_8_; (**b**) Ce_Sr1_Li_Sr2_-Sr_2_Si_5_N_8_; (**c**) Ce_Sr2_Li_Sr1_-Sr_2_Si_5_N_8_; (**d**) Ce_Sr2_Li_Sr2_-Sr_2_Si_5_N_8_; (**e**) Pr_Sr1_Li_Sr1_-Sr_2_Si_5_N_8_; (**f**) Pr_Sr1_Li_Sr2_-Sr_2_Si_5_N_8_; (**g**) Pr_Sr2_Li_Sr1_-Sr_2_Si_5_N_8_; (**h**) Pr_Sr2_Li_Sr2_-Sr_2_Si_5_N_8_; (**i**) Tb_Sr1_Li_Sr1_-Sr_2_Si_5_N_8_; (**j**) Tb_Sr1_Li_Sr2_-Sr_2_Si_5_N_8_; (**k**) Tb_Sr2_Li_Sr1_-Sr_2_Si_5_N_8_; (**l**) Eu_Sr1_-Sr_2_Si_5_N_8_; (**m**) Eu_Sr2_-Sr_2_Si_5_N_8_.

The direct bandgaps of Figure 8a–c are 0.51 eV, 0.78 eV, 0.87 eV, 0.57 eV; 0.39 eV, 0.18 eV, 0.18 eV, 0.27 eV; 1.98 eV, 2.07 eV, 2.01 eV, respectively. The U_eff_ value makes 4f-CBM fall in the appropriate energy range, the U_eff_ introduced by (a–k) are: 5 eV, 5 eV, 5 eV, 6 eV; 4 eV, 6 eV, 6 eV, 6 eV; 2 eV, 2 eV, 2 eV, 2 eV. The energy difference of 4f-CBM in (a–k) with different U_eff_ is: 2.42 eV, 2.53 eV, 2.62 eV, 2.68 eV, 1.79 eV, 2.28 eV, 2.28 eV, 2.37 eV; 2.69 eV, 2.76 eV, 2.73 eV.

In summary, we add different U_eff_ to the strongest peak of the 4f energy level and the energy distribution range in Figure 8a–k to make it fall within the appropriate range. The energy ranges of 4f-CBM in Figure 8a–d are 2.42–2.85 eV, 2.80–3.03 eV, 2.65–3.13 eV, 2.67–2.91 eV, respectively. In Figure 8a, Ce_Sr1_Li_Sr1_-Sr_2_Si_5_N_8_ is closer to the excitation energy range of Ce^3+^ doped Sr_2_Si_5_N_8_ from 2.85 to 3.25 eV [30] reported in the experiment, but Figure 8a has a global redshift of 0.43 eV. In Figure 7e–h, the energy ranges of 4f-CBM are 2.75–2.99 eV, 2.28–3.06 eV, 2.27–3.08 eV, 2.37–2.97 eV, respectively. In Figure 8e, Pr_Sr1_Li_Sr1_-Sr_2_Si_5_N_8_ is closer to the excitation energy range of Pr^3+^ doped SrAl_2_O_4_ from 2.53 to 2.88 eV [30] reported in the experiment. The energy ranges of 4f-CBM in Figure 8i–k are 2.68–3.04 eV, 2.77–3.07 eV, 2.71–3.07 eV, respectively, which is far from the experimental excitation of Sr_2_Si_5_N_8_:Tb^3+^ [16]. The U_eff_ values of Eu^2+^ and Ce^3+^ are 4 eV and 6 eV, respectively, and the energy range is mainly 2.27–2.82 eV, and the peak value is 2.27 eV, 2.39 eV, 2.82 eV. In Figure S2b, the Eu^2+^ 4f energy level is not in the forbidden band. When Ce U_eff_ is 5 eV, the energy range of 4f-CBM is from 2.35 to 2.83 eV. The Eu^2+^, Pr^3+^ U_eff_ values in Figure S2c are, respectively, 1 eV, 7 eV, and Eu^2+^ 4f energy levels have three strong peaks of 2.19 eV, 2.25 eV, and 2.93 eV. Pr^3+^ has the highest peak intensity of 2.69 eV in 2.69–2.99 eV.

## 3. Material and Methods

### 3.1. Theoretical Models

**Figure 9:**
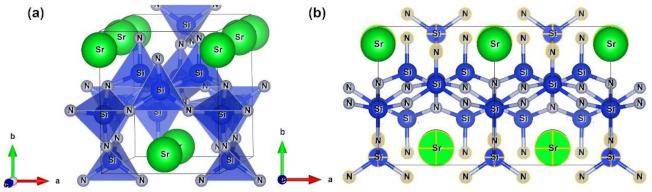
(**a**) Molecular structure of Sr_2_Si_5_N_8_; (**b**) 2 × 2 × 1 supercell of Sr_2_Si_5_N_8_: The selected atoms are Sr_2_.

Three lanthanide ions (Ln^3+^ = Ce^3+^, Pr^3+^, Tb^3+^) were selected as doping ions to dope Sr_2_Si_5_N_8_ and Sr_2_Si_5_N_8_:Eu^2+^, respectively. As shown in Figure 9a, there are two kinds of Sr doping sites, namely, eight-coordinate Sr_1_ [SrN_8_] (0.5000, 0.8734, 0.9997) and ten-coordinate Sr_2_ [SrN_10_] (0.7500, 0.1158, 0.8683). Since in the doped ions, Ln are all positively trivalent and Sr is bivalent to neutralize the entire system’s charge, every time a positive trivalent lanthanide ion is introduced to replace Sr^2+^, a Li^+^ is introduced to replace Sr^2+^ to keep the entire system electrically neutral. The chemical formula of Sr_2_Si_5_N_8_ doped with Ln^3+^/Li^+^ is Ln_Sr1/Sr2_Li_Sr1/Sr2_Sr_2_Si_5_N_8_, while the chemical formula of Sr_2_Si_5_N_8_ doped with Ln^3+^/Li^+^/Eu^2+^ is Ln_Sr1/Sr2_Eu_Sr1/Sr2_Li_Sr1/Sr2_-Sr_2_Si_5_N_8_. Figure 9b has established a 2 × 2 × 1 supercell (60 atoms) with a doping concentration of 12.5% for Ln^3+^, Eu^2+^, and Li^+^. If we continue to expand the unit cell to 3 × 2 × 1 to reduce the doping concentration, the calculation requires more K points than 5 × 8 × 6, and it is complicated for the structure to converge. Notably, 2 × 2 × 1 is the largest supercell structure that can be established under the premise that the structure can converge.

### 3.2. Computational Methods

When considering all ground-state calculations, density functional theory (DFT) calculations are performed in the Vienna AB Initio Simulation Package (VASP, Vienna ab initio simulation package) using the projector-augmented wave (PAW) method [31]. Exchange-correlation (XC, exchange-correlation) energy is described in the Perdew–Burke–Ernzerhof (PBE) method in generalized gradient approximation (GGA) [32]. The cutoff energy of all calculated plane wave bases is set to 500 eV. The energy convergence tolerance of the Sself-Cconsistent Ffield (SCF) is 10^−4^ eV. The convergence tolerance of the relaxation force is 0.01 eV/Å per atom. Sr_2_Si_5_N_8_:Eu^2+^ original unit cell model removed the energy band calculation.

To solve the problem that DFT cannot handle d and f electrons, the Hubbard model compensates for the strong correlation between d and f electrons by adding additional energy terms. The corrected energy’s form is as follows:

(3) $$E^{DFT+U}=E^{DFT}+\frac{1}{2}\underset{I,\sigma }{\sum }\underset{nl}{\sum }U_{nl}^{I}\left[\left(n_{nl}^{I\sigma }\left(1-n_{nl}^{I\sigma }\right)\right)\right]$$

There are many forms of Hubbard model correction. We choose the simplest Dudarev approximation [33]; the form is as follows:

(4) $$\frac{\left(U-J\right)}{2}\underset{\sigma }{\sum }\left[\left(\underset{m1}{\sum }n_{m1,m1}^{\sigma }\right)-\left(\underset{m1,m2}{\sum }{\hat{n}}_{m1,m2}^{\sigma }{\hat{n}}_{m2,m1}^{\sigma }\right)\right]$$

*U* and *J* are the critical parameters of Hubbard’s correction item, replaced by = (*U* − *J*). Different ions have different U_eff_ values in different host environments.

In the whole calculation process, a 5 × 8 × 6 *k*-point network is generated using the Monkhorst–Pack method with Γ as the center. In the calculation of the energy band structure, the high-symmetry K point and the appropriate reverse spatial path are determined according to the symmetry of the crystal lattice. The electronic configuration 3s^2^3p^2^, 2s^2^p^3^, 4s^2^4p^6^5s^2^, 2s^1^, 5s^2^5p^6^6s^2^4f^7^, 5s^2^5p^6^4f^1^5d^1^6s^2^, 5s^2^6s^2^5p^6^5d^1^4f^2^, and 4f^8^5s^2^6s^2^5p^6^5d^1^ correspond to Si, N, Sr, Li, Eu, Ce, Pr, and Tb in the pseudo-electron composition.

Regarding the calculation method of the effective coordination number, this article adopts Brunner’s method [34], which assumes that ionic or covalent bonds connect the central atoms of the surrounding atoms. In the established *C.N* principle, the energy standard is defined as each coordination. The bond energy between (*X_i_*) and the central atom (*M*) is different from the bond energy between the nearest ligand (*X*_0_) and the central atom. The energy ratio $E_{\left(M-X_{i}\right)}$:$\;E_{\left(M-X_{0}\right)}$ is defined as the contribution of *X_i_* atom to *M* atom C.N*. If the nearest atom is only affected by the Coulomb force, it is easy to get formula (5)

(5) $$C.N_{ion}^{*}=\underset{i}{\sum }E_{\left(M-X_{i}\right)}/E_{\left(M-X_{0}\right)}=\underset{i}{\sum }\Upsilon_{\left(M-X_{0}\right)}/\Upsilon_{\left(M-X_{i}\right)}$$

where $\Upsilon_{\left(M-X_{i}\right)}$ is the bond length between the central atom and the ligand.

The formation of energy can evaluate the stability of the structure. The model after substitution and doping lattice can be described as the following formula (6):

*E*_f_(Sr_2_Si_5_N_8_:Eu^2+^/Ln^3+^/Li^+^) = *E*(Sr_2_Si_5_N_8_:Eu^2+^/Ln^3+^/Li^+^)^+^3*µ*(Sr^2+^) − *E*(Sr_2_Si_5_N_8_) − *µ*(Eu^2+^) − *µ*(Ln^3+^) − *µ*(Li^+^) (6)

In the above formula *E*_f_(Sr_2_Si_5_N_8_:Eu^2+^/Ln^3+^/Li^+^) is the formation energy, *E*(Sr_2_Si_5_N_8_:Eu^2+^/Ln^3+^/Li^+^) is the energy calculated by SCF, *µ*(Sr^2+^), *µ*(Eu^2+^), *µ*(Li^+^) are the chemical potentials of Sr, Eu, Li, and Ln, respectively, and *E*(Sr_2_Si_5_N_8_) is the energy calculated by the lattice matrix SCF. For the chemical potential of the element, formula (7) can be used

*µ*(*x*) = *E*(Cell of *X*)/*n* (7)

where *µ*(*x*) is the chemical potential of *X*, *E* (Cell of *X*) is the elemental unit cell of element *X*, and *n* is the number of *X* contained in the elemental unit cell.

## 4. Conclusions

In this study, two models of Sr_2_Si_5_N_8_:Ln^3+^/Li^+^ and Sr_2_Si_5_N_8_:Ln^3+^/Eu^2+^/Li^+^ were constructed. The calculation of Sr_2_Si_5_N_8_:Ln^3+^/Li^+^ and Sr_2_Si_5_N_8_:Eu^2+^ provides a basic reference for double-doped lanthanide ions. The introduction of Li^+^ and Ln^3+^ can achieve the design idea of neutralizing electrons and maintaining the original structure. Three dual-doped models of Eu_Sr1_Ce_Sr1_Li_Sr2_-Sr_2_Si_5_N_8_, Eu_Sr2_Ce_Sr1_Li_Sr2_-Sr_2_Si_5_N_8_, and Eu_Sr2_Pr_Sr2_Li_Sr2_-Sr_2_Si_5_N_8_ were screened out. Among the three models mentioned, Eu_Sr1_Ce_Sr1_Li_Sr2_-Sr_2_Si_5_N_8_ and Eu_Sr2_Pr_Sr2_Li_Sr2_-Sr_2_Si_5_N_8_’s fluorescence performances are expected to exceed that of Sr_2_Si_5_N_8_:Eu^2+^, which can be regarded as a potential route to increase the quenching temperature of the phosphor. When Ce^3+^ U_eff_ in Ce_Sr1_Li_Sr1_-Sr_2_Si_5_N_8_ is 5 eV, the energy range of 4f-CBM is 2.42 –2.85 eV. Compared with the experimentally reported excitation range of 2.85–3.25 eV, the redshift is 0.42 eV (75 nm). When Pr^3+^ U_eff_ in Pr_Sr1_Li_Sr1_-Sr_2_Si_5_N_8_ is 4 eV, the energy range of 4f-CBM is 2.75–2.99 eV, and its peak value is 2.90 eV, which is similar to SrAl_2_O_4_:Pr^3+^ 430–490nm with peaks around 440 nm. In Sr_2_Si_5_N_8_:Ln^3+^/Li^+^/Eu^2+^, three convergent models of Eu_Sr1_Ce_Sr1_Li_Sr1_-Sr_2_Si_5_N_8_, Eu_Sr2_Ce_Sr1_Li_Sr1_-Sr_2_Si_5_N_8_, and Eu_Sr2_Pr_Sr2_Li_Sr1_-Sr_2_Si_5_N_8_ were selected. The addition of Ce^3+^ in Eu_Sr1_Ce_Sr1_Li_Sr1_-Sr_2_Si_5_N_8_ made the 4f energy level of Eu^2+^ blue shift. Similarly, the addition of Pr^3+^ in Eu_Sr2_Pr_Sr2_Li_Sr1_-Sr_2_Si_5_N_8_ makes part of the Eu^2+^ 4f energy level blue shift. The Eu^2+^ 4f energy level in Eu_Sr2_Ce_Sr1_Li_Sr1_-Sr_2_Si_5_N_8_ is not in the forbidden band, so Eu^2+^ is not used as the emission center.

## Figures and Tables

**Table 1:** Cell parameters and [LnN] polyheral structures of Sr_2_Si_5_N_8_ and Sr_2_Si_5_N_8_ doped by Ln (Ce^3+^, Pr^3+^, Tb^3+^)/Li^+^, Eu^2+^.

2 × 2 × 1	Cell Parameters				Volume (Å^3)^	[LnN] Polyhedral Volume (Å^3^)	[LnN] Distortion Index (Å)	[LnN] Effective Coordination Number
Supercells	a (Å)	b (Å)	c (Å)	α,β,γ/(°)				
Sr_2_Si_5_N_8_(Sr_2_)	11.498	6.881	9.405	α = β = γ = 90.0	744.074	48.866	0.062	6.707
Sr_2_Si_5_N_8_(Sr_1_)	11.498	6.881	9.405	α = β = γ = 90.0	744.074	32.657	0.075	5.448
Eu_Si2_-Sr_2_Si_5_N_8_	11.498	6.876	9.403	α = 89.9, β = γ = 90.0	743.131	48.545	0.072	5.771
Eu_Sr1_-Sr_2_Si_5_N_8_	11.493	6.876	9.402	α = 90.1, β = γ = 90.0	743.046	32.379	0.090	4.728
Ce_Sr1_Li_Sr1_-Sr_2_Si_5_N_8_	11.492	6.874	9.373	α = 90.4, β = γ = 90.0	740.450	27.921	0.119	4.919
Ce_Sr1_Li_Sr2_-Sr_2_Si_5_N_8_	11.505	6.869	9.376	α = 90.1, β = γ = 90	741.010	27.857	0.110	4.776
Ce_Sr2_Li_Sr1_-Sr_2_Si_5_N_8_	11.485	6.876	9.370	α = 89.9, β = γ = 90	739.910	45.191	0.092	5.550
Ce_Sr2_Li_Sr2_-Sr_2_Si_5_N_8_	11.504	6.863	9.380	α = 89.7, β = γ = 90	740.700	44.354	0.090	5.597
Pr_Sr1_Li_Sr1_-Sr_2_Si_5_N_8_	11.487	6.873	9.372	α = 90.4, β = γ = 90	739.830	27.797	0.123	4.849
Pr_Sr1_Li_Sr2_-Sr_2_Si_5_N_8_	11.501	6.872	9.372	α = 90.1, β = γ = 90.0	740.680	27.689	0.114	4.645
Pr_Sr2_Li_Sr1_-Sr_2_Si_5_N_8_	11.498	6.881	9.405	α = β = γ = 90.0	739.340	45.213	0.101	5.420
Pr_Sr2_Li_Sr2_-Sr_2_Si_5_N_8_	11.502	6.863	9.380	α = 89.7, β = γ = 90.0	740.450	44.345	0.093	5.337
Tb_Sr1_Li_Sr1_-Sr_2_Si_5_N_8_	11.470	6.863	9.360	α = 90.5, β = γ = 90.0	736.970	27.321	0.154	4.676
Tb_Sr1_Li_Sr2_-Sr_2_Si_5_N_8_	11.493	6.859	9.370	α = 90.1, β = γ = 90.0	738.970	27.300	0.147	4.496
Tb_Sr2_Li_Sr1_-Sr_2_Si_5_N_8_	11.479	6.850	9.376	α = 89.9, β = γ = 90.0	737.230	44.902	0.150	4.724

**Table 2:** [SrN] Coordination polyhedron parameters of Sr_2_Si_5_N_8_ doped by Ln (Ce^3+^, Pr^3+^, Tb^3+^)/Li^+^.

2 × 2 × 1 Supercell	[SrN] Average Bond Length (Å)	[SrN] Polyhedral Volume (Å^3^)	[SrN] Distortion Index (Å)	[SrN] Effective Coordination Number	[SrN] Coordination Number
Ce_Sr1_Li_Sr1_-Sr_2_Si_5_N_8_	2.994	49.700	0.0723	7.000	10
	2.942	48.317	0.0720	6.620	10
	2.886	32.405	0.0757	5.420	8
Ce_Sr1_Li_Sr2_-Sr_2_Si_5_N_8_	2.925	47.655	0.063	7.070	10
	2.954	48.845	0.073	6.358	10
	2.907	35.144	0.086	5.052	8
Ce_Sr2_Li_Sr1_-Sr_2_Si_5_N_8_	2.956	49.440	0.066	6.211	10
	2.963	45.359	0.088	5.889	10
	2.879	32.349	0.081	5.741	8
Ce_Sr2_Li_Sr2_-Sr_2_Si_5_N_8_	2.975	49.291	0.075	5.989	10
	2.870	34.093	0.080	5.350	8
	2.909	33.711	0.067	5.899	8
Pr_Sr1_Li_Sr1_-Sr_2_Si_5_N_8_	2.940	48.289	0.072	6.646	10
	2.994	49.734	0.073	7.022	10
	2.884	32.335	0.076	5.434	8
Pr_Sr1_Li_Sr2_-Sr_2_Si_5_N_8_	2.923	47.601	0.062	7.148	10
	2.956	48.950	0.072	6.378	10
	2.907	35.144	0.085	5.085	8
Pr_Sr1_Li_Sr2_-Sr_2_Si_5_N_8_	2.923	47.601	0.062	7.148	10
	2.907	35.144	0.085	5.085	8
	2.955	48.950	0.072	6.378	10
Pr_Sr2_Li_Sr2_-Sr_2_Si_5_N_8_	2.970	49.192	0.073	6.087	10
	2.872	34.160	0.081	5.321	8
	2.908	33.695	0.068	5.886	8
Tb_Sr1_Li_Sr1_-Sr_2_Si_5_N_8_	2.937	48.233	0.071	6.874	10
	2.999	49.821	0.075	6.968	10
	2.877	32.096	0.075	5.467	8
Tb_Sr1_Li_Sr2_-Sr_2_Si_5_N_8_	2.923	47.602	0.061	7.231	10
	2.963	49.304	0.073	6.116	10
	2.899	34.905	0.083	5.159	8
Tb_Sr2_Li_Sr1_-Sr_2_Si_5_N_8_	2.883	32.733	0.086	5.729	8
	2.958	49.914	0.079	5.940	10
	2.955	45.191	0.085	6.112	10
Eu_Sr1_-Sr_2_Si_5_N_8_	2.947	48.690	0.061	6.735	10
	2.952	48.914	0.060	6.962	10
	2.888	32.658	0.074	5.449	8
Eu_Sr2_-Sr_2_Si_5_N_8_	2.951	48.904	0.061	6.701	10
	2.888	32.706	0.072	5.625	8
	2.889	32.641	0.074	5.453	8
	2.942	38.657	0.085	5.536	8

**Table 3:** Cell parameters and [LnN] polyheral structures of Sr_2_Si_5_N_8_ co-doped Eu^2+^/Ce^3+^/Li^+^ and Eu^2+^/Pr^3+^/Li^+^.

2 × 2 × 1	Cell Parameters				Volume (Å^3^)	[LnN] Polyhedral Volume (Å^3^)	[LnN] Distortion Index (Å)	[LnN] Effective Coordination Number
Supercells	a (Å)	b (Å)	c (Å)	α,β,γ (°)				
Eu_Sr1_Ce_Sr1_Li_Sr2_-Sr_2_Si_5_N_8_(Eu)	11.490	6.874	9.375	α = 90.3, β = γ = 90.0	740.470	32.042	0.0947	4.654
Eu_Sr1_Ce_Sr1_Li_Sr2_-Sr_2_Si_5_N_8_(Ce)	11.490	6.874	9.375	α = 90.3, β = γ = 90.0	740.470	28.112	0.1219	4.926
Eu_Sr2_Ce_Sr1_Li_Sr2_-Sr_2_Si_5_N_8_(Eu)	11.492	6.867	9.370	α = 90.3, β = γ = 90.0	739.470	48.094	0.0613	6.455
Eu_Sr2_Ce_Sr1_Li_Sr2_-Sr_2_Si_5_N_8_(Ce)	11.492	6.867	9.370	α = 90.3, β = γ = 90.0	739.470	28.046	0.1254	5.026
Eu_Sr2_Pr_Sr2_Li_Sr2_-Sr_2_Si_5_N_8_(Eu)	11.480	6.8607	9.377	α = 90.3, β = γ = 90.0	738.566	48.574	0.0943	5.849
Eu_Sr2_Pr_Sr2_Li_Sr2_-Sr_2_Si_5_N_8_(Pr)	11.480	6.8607	9.377	α = 90.3, β = γ = 90.0	738.566	45.876	0.0880	4.752

**Table 4:** [SrN] Coordination polyhedron parameters of Sr_2_Si_5_N_8_ doped by Ln (Ce^3+^, Pr^3+^, Tb^3+^)/Li^+^/Eu^2+^.

2 × 2 × 1 Supercell	[SrN] Average Bond Length (Å)	[SrN] Polyhedral Volume (Å^3^)	[SrN] Distortion Index (Å)	[SrN] Effective Coordination Number	[SrN] Coordination Number
Eu_Sr1_Ce_Sr1_Li_Sr1_	2.941	48.361	0.0666	6.884	10
	2.989	49.546	0.0721	7.040	10
	2.887	32.507	0.0755	5.406	8
Eu_Sr2_Ce_Sr11_Li_Sr1_	2.945	48.444	0.0718	6.590	10
	2.991	49.691	0.0705	7.039	10
	2.880	32.241	0.0755	5.427	8
Eu_Sr2_Pr_Sr21_Li_Sr1_	2.960	49.248	0.0646	6.260	10
	2.863	31.895	0.0825	5.680	8
	2.889	32.623	0.0753	5.360	8

**Table 5:** Energy level difference of Eu 4f-CBM of Sr_2_Si_5_N_8_:Eu^2+^ with different Ueff parameters.

U_eff_ (eV)	Eu^2+^ 4f—CBM(eV)	
	Eu_1_^2+^	Eu_2_^2+^
0	0.84	0.84
2	1.48	1.50
4	2.22	2.23
6	2.86	2.93

## Data Availability

The data that support the findings of this study are available from the corresponding authors upon reasonable request.

## Supplementary Materials

The following are available online. Figure S1: Densiy of states of doped structures: (a) CeSr_1_LiSr_1_-Sr_2_Si_5_N_8_; (b) CeSr_1_LiSr_2_-Sr_2_Si_5_N_8_; (c) CeSr_2_LiSr_1_-Sr_2_Si_5_N_8_; (d) CeSr_2_LiSr_2_-Sr_2_Si_5_N_8_; (e) PrSr_1_LiSr_1_-Sr_2_Si_5_N_8_; (f) PrSr_1_LiSr_2_-Sr_2_Si_5_N_8_; (g) PrSr_2_LiSr_1_-Sr_2_Si_5_N_8_; (h) PrSr_2_LiSr_2_-Sr_2_Si_5_N_8_; (i) TbSr_1_LiSr_1_-Sr_2_Si_5_N_8_; (j) TbSr_1_LiSr_2_-Sr_2_Si_5_N_8_; (k) TbSr_1_LiSr_2_-Sr_2_Si_5_N_8_; (l) EuSr_1_-Sr_2_Si_5_N_8_; (m) EuSr_2_-Sr_2_Si_5_N_8_. Figure S2: Densiy of states of doped structures: (a) EuSr_1_CeSr_1_LiSr_2_-Sr_2_Si_5_N_8_; (b) EuSr_2_CeSr_1_LiSr_2_-Sr_2_Si_5_N_8_; (c) PrSr_2_EuSr_2_LiSr_2_-Sr_2_Si_5_N_8_. Table S1: The Key information in the energy band structure of Sr_2_Si_5_N_8_:Ln^3+^/Li^+^, Eu^2+^. Table S2: The Key information in the energy band structure of Sr_2_Si_5_N_8_:Ln^3+^/Li^+^/Eu^2+^.

## Sample Availability

The samples of compounds are not available from authors.
