# Supplementary material for: Research on Molecular Structure and Electronic Properties of Ln3+ (Ce3+, Tb3+, Pr3+)/Li+ and Eu2+ Co-Doped Sr2Si5N8 via DFT Calculation
Source: Molecules. 2021 Mar 25;26(7):1849. doi: 10.3390/molecules26071849 (PMC8037467; doi:10.3390/molecules26071849)
Supplement: Supplementary file 1 [file molecules-26-01849-s001.pdf]

Supporting Information

Research on Molecular Structure and Electronic Properties of Ln^{3+} (Ce^{3+}, Tb^{3+}, Pr^{3+}) /Li^{+} and Eu^{2+} Co-doped $Sr_2Si_5N_8$ via DFT Calculation

Ziqian Yin[1], Meijuan Li[1,2*], Jianwen Zhang[1] and Qiang Shen[1*]

[1] State Key Laboratory of Advanced Technology for Materials Synthesis and Processing, Wuhan University of Technology, Wuhan 430070, China

[2] School of Chemistry, Chemical Engineering and Life Sciences, Wuhan University of Technology, Wuhan 430070, China

*Corresponding author: Meijuan Li: A.P.; Ph.D.; E-mail: meijuanli@whut.edu.cn; Qiang Shen: Prof.; Ph.D.; E-mail: sqqf@263.net

Table.S1 The Key information in the energy band structure of $Sr_2Si_5N_8$: Ln^{3+}/Li^{+}, Eu^{2+}

2x2x1 Supercells	CBM (eV)	High symmetry point where CBM is located	VBM (eV)	High symmetry point where CBM is located	Each energy level distribution (eV)
$Ce_{Sr1}Li_{Sr1}$-$Sr_2Si_5N_8$	3.03	G	0	Z-G	2.04, 2.13, 2.28, 2.31, 2.46, 2.52
$Ce_{Sr1}Li_{Sr2}$-$Sr_2Si_5N_8$	3.15	G	0	Z-G	2.07, 2.10, 2.25, 2.34, 2.37
$Ce_{Sr2}Li_{Sr1}$-$Sr_2Si_5N_8$	3.24	Z	0	Z-G	1.86, 2.01, 2.31, 2.37
$Ce_{Sr2}Li_{Sr2}$-$Sr_2S_{i5}N_8$	3.09	G	0	Z-G	2.25, 2.43, 2.49, 2.52
$Pr_{Sr1}Li_{Sr1}$-$Sr_2Si_5N_8$	2.97	G	0	Z-G	1.38, 1.53, 1.62, 2.58
$Pr_{Sr1}Li_{Sr2}$-$Sr_2Si_5N_8$	3.27	G	0	Z-G	2.28, 2.31, 3.06, 3.09
$Pr_{Sr2}Li_{Sr1}$-$Sr_2Si_5N_8$	3.27	G	0	Z-G	2.28, 2.31, 3.06, 3.09
$Pr_{Sr2}Li_{Sr2}$-$Sr_2Si_5N_8$	3.30	Z	0	Z-G	2.43, 2.52, 2.88, 3.03
$Tb_{Sr1}Li_{Sr1}$-$Sr_2Si_5N_8$	3.30	Z	0	Z-G	0.87, 1.23, 1.32
$Tb_{Sr1}Li_{Sr2}$-$Sr_2Si_5N_8$	3.27	Z	0	Z-G	0.87, 0.96, 1.20
$Tb_{Sr2}Li_{Sr1}$-$Sr_2Si_5N_8$	3.24	Z	0	Z-G	0.87, 1.14
Eu_{Si1}-$Sr_2Si_5N_8$	3.18	Z	0	Z-G	2.25, 2.37
Eu_{Si2}-$Sr_2Si_5N_8$	3.21	Z	0	Z-G	2.22,2.34

Table.S2 The Key information in the energy band structure of $Sr_2Si_5N_8$: $Ln^{3+}/Li^{+}/Eu^{2+}$

2x2x1 Supercells	CBM (eV)	High symmetry point where CBM is located	VBM (eV)	High symmetry point where CBM is located	Each energy level distribution(eV)
$Eu_{Sr1}Ce_{Sr1}Li_{Sr1}$-$Sr_2Si_5N_8$	3.15	G	0	Z-G	1.77, 1.89, 2.31, 2.64, 2.76, 2.79, 2.82, 2.88
$Eu_{Sr2}Ce_{Sr1}Li_{Sr1}$-$Sr_2Si_5N_8$	3.03	G	0	Z-G	1.97, 1.98, 2.01, 2.10, 2.22, 2.40, 2.70
$Eu_{Sr2}Pr_{Sr2}Li_{Sr1}$-$Sr_2Si_5N_8$	3.15	G	0	Z-G	0.57, 1.32, 2.61, 2.91, 3.15, 3.18

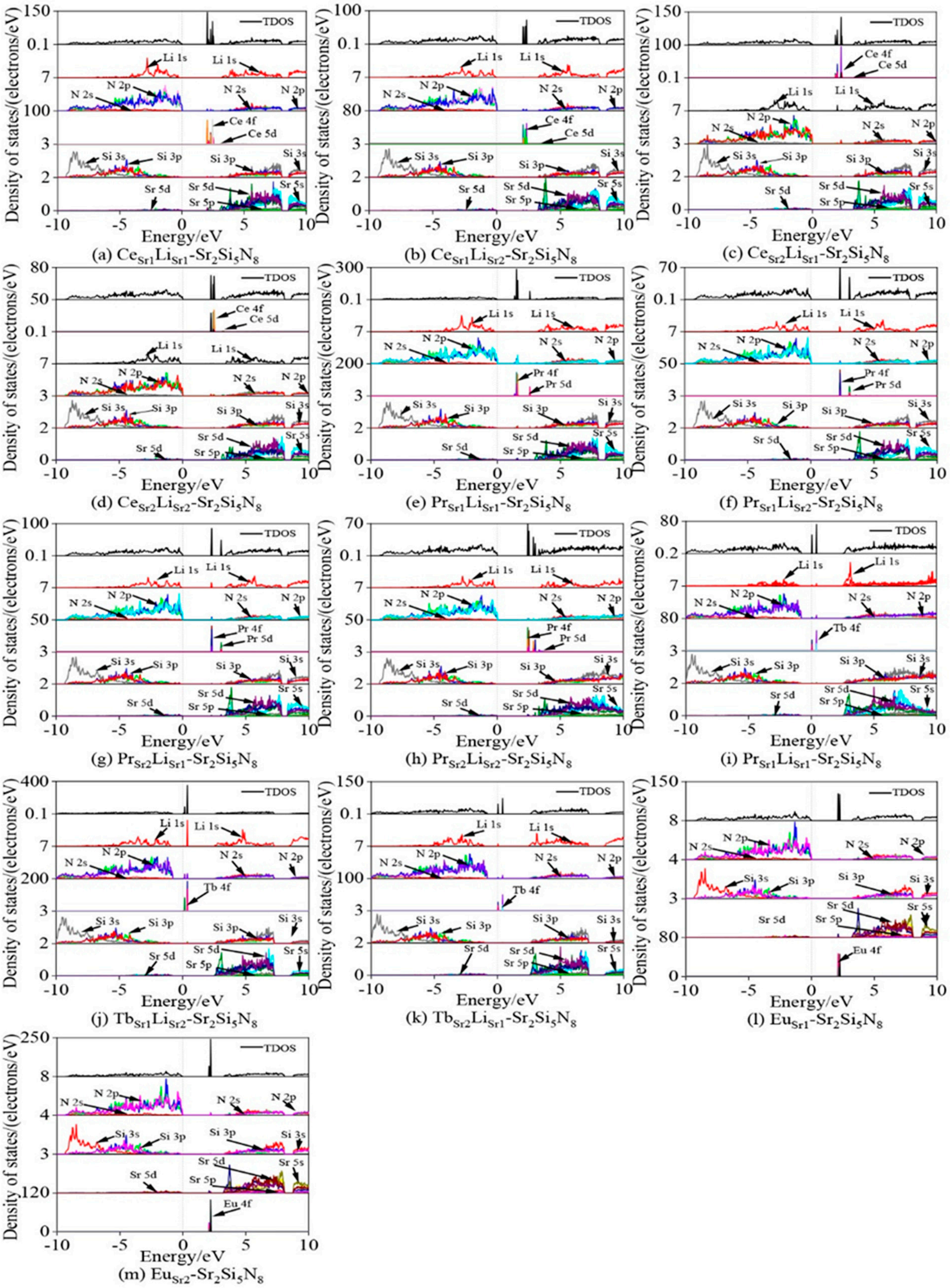

Fig.S1 Densiy of states of doped structures: (a) $Ce_{Sr1}Li_{Sr1}$-$Sr_2Si_5N_8$; (b) $Ce_{Sr1}Li_{Sr2}$-$Sr_2Si_5N_8$; (c) $Ce_{Sr2}Li_{Sr1}$-$Sr_2Si_5N_8$; (d) $Ce_{Sr2}Li_{Sr2}$-$Sr_2Si_5N_8$; (e) $Pr_{Sr1}Li_{Sr1}$-$Sr_2Si_5N_8$; (f) $Pr_{Sr1}Li_{Sr2}$-$Sr_2Si_5N_8$; (g) $Pr_{Sr2}Li_{Sr1}$-$Sr_2Si_5N_8$; (h) $Pr_{Sr2}Li_{Sr2}$-$Sr_2Si_5N_8$; (i) $Tb_{Sr1}Li_{Sr1}$-$Sr_2Si_5N_8$; (j) $Tb_{Sr1}Li_{Sr2}$-$Sr_2Si_5N_8$; (k) $Tb_{Sr1}Li_{Sr2}$-$Sr_2Si_5N_8$; (l) Eu_{Sr1}-$Sr_2Si_5N_8$; (m) Eu_{Sr2}-$Sr_2Si_5N_8$.

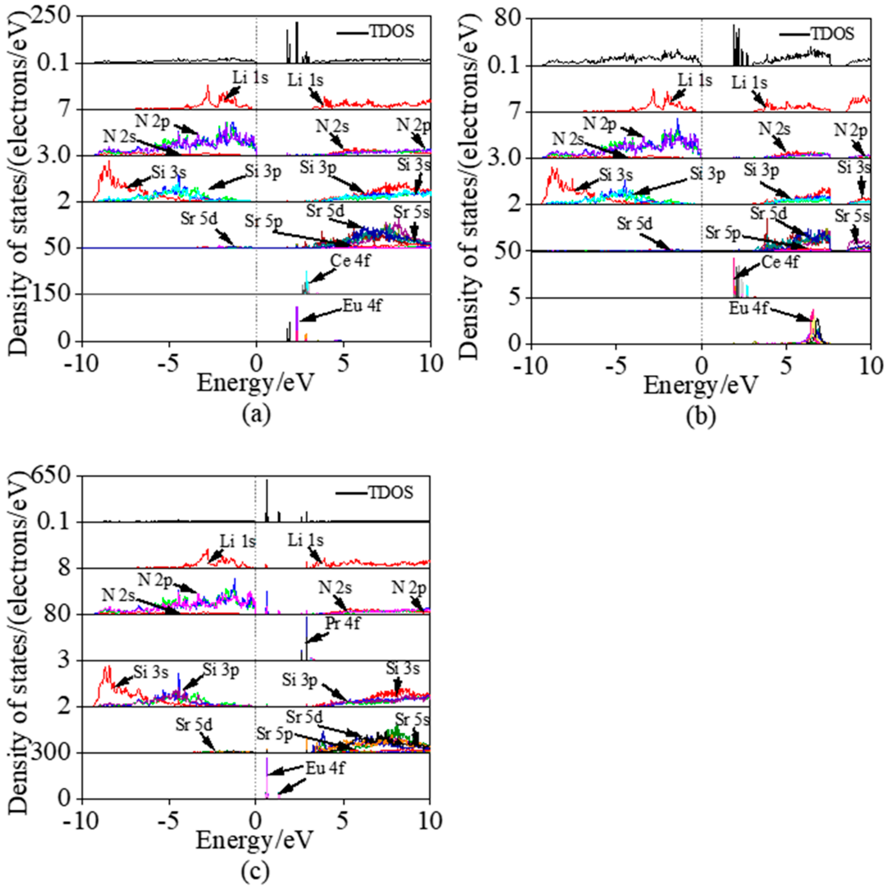

Fig.S2 Densiy of states of doped structures: (a) $Eu_{Sr1}Ce_{Sr1}Li_{Sr2}$-$Sr_2Si_5N_8$; (b) $Eu_{Sr2}Ce_{Sr1}Li_{Sr2}$-$Sr_2Si_5N_8$; (c) $Pr_{Sr2}Eu_{Sr2}Li_{Sr2}$-$Sr_2Si_5N_8$
